# Turn on Fluorescent Probes for Selective Targeting of Aldehydes

**DOI:** 10.3390/chemosensors4010005

**Published:** 2016-03-11

**Authors:** Ozlem Dilek, Susan L. Bane

**Affiliations:** 1 Istanbul Kemerburgaz University, School of Medicine, Department of Medical Biochemistry, Bagcilar, Istanbul 34217, Turkey; 2 Department of Chemistry, State University of New York at Binghamton, Binghamton, NY 13902, USA

**Keywords:** fluorescence, amine, imine, aldehyde, fluorescent probes

## Abstract

Two different classes of fluorescent dyes were prepared as a turn off/on sensor system for aldehydes. Amino derivatives of a boron dipyrromethene (BDP) fluorophore and a xanthene-derived fluorophore (rosamine) were prepared. Model compounds of their product with an aldehyde were prepared using salicylaldehyde. Both amino boron dipyrromethene and rosamine derivatives are almost non-fluorescent in polar and apolar solvent. However, imine formation with salicylaldehyde on each fluorophore increases the fluorescence quantum yield by almost a factor of 10 (from 0.05 to 0.4). These fluorophores are therefore suitable candidates for development of fluorescence-based sensors for aldehydes.

## 1. Introduction

Aldehydes are essential for production of synthetic resins, synthetic dyes, flavorings, perfumes, and other chemicals. They are widely used as disinfectants and preservatives in many settings. As a result, aldehydes are frequently released to the environment, and their presence needs to be monitored [1]. Methods to monitor aldehydes include simple colorometric measurements [2], and more involved electrochemical, gas chromatography, and chemiluminiscent techniques [4]. One common approach is to detect the product of the reaction of an aldehyde with an aromatic hydrazine. Covalent bond formation is monitored by changes in optical properties such as absorption and fluorescence spectra [1]. Since hydrazines are considered to be an environmental hazard [1,2], potentially less toxic reagents should be considered. Reactions with these reagents should retain the speed and ease of the hydrazine reaction, however, and preferably a sensitive optical signal such as fluorescence.

**Figure 1.**
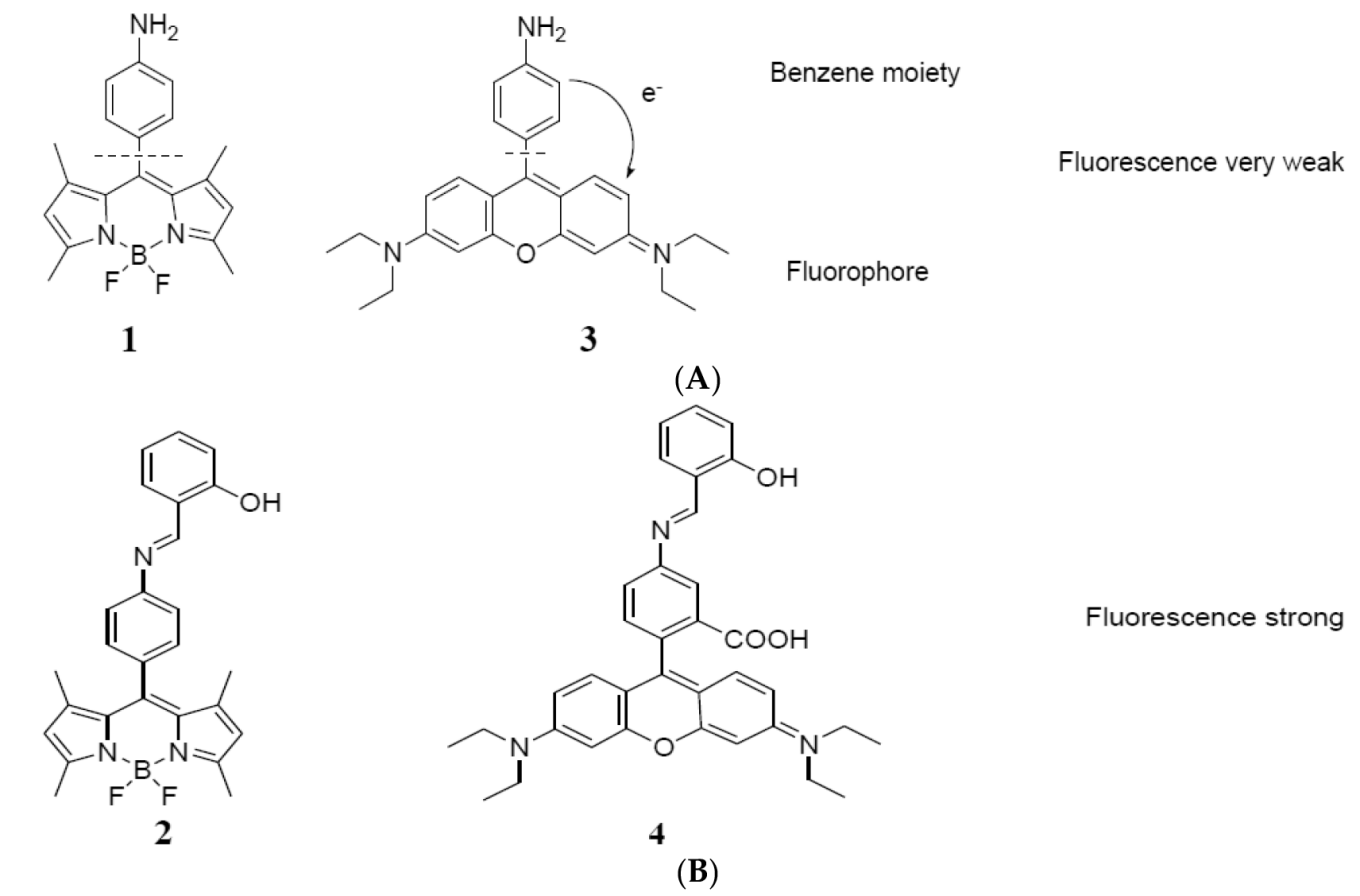
(**A**) structure of amine derivatives of BDP and rosamine. Electron transfer from the benzene moiety to the fluorophore. (**B**) structure of imine derivatives of BDP and rosamine.

In this study, we report about two fluorophores that are suitable for developing a simple, rapid and off/on fluorescence switching method. The selection of the target molecules is based on an expected change in the optical properties of the probe based on a photoelectron transfer (PeT) mechanism of fluorescence quenching. We noted that Munkholm previously reported that fluoresceinamine has a quenched fluorescence due to amine group, but conversion of amine to an amide restored the full fluorescent indicator properties of fluorescein [8]. Gabe and his coworkers previously reported some computational studies [6] on calculation of the HOMO (highest occupied molecular orbital) energy level of different PeT donors for the boron dipyrromethene (BDP) chromophore and noted a threshold value for the HOMO that would result in quenched fluorescence. It therefore seemed reasonable that affecting the electron density on the amine by imine formation might also yield an increase in BDP fluorescence and possible other classes of fluorophores such as the xanthenes (Figure 1). In this report, we confirm this hypothesis using synthetic model compounds. Therefore, turn off/on amine/imine fluorescent dyes have potential uses as sensors in for selective detection or monitoring of aldehydes.

## 2. Results and Discussion

### 2.1. Synthesis

**Scheme 1.**
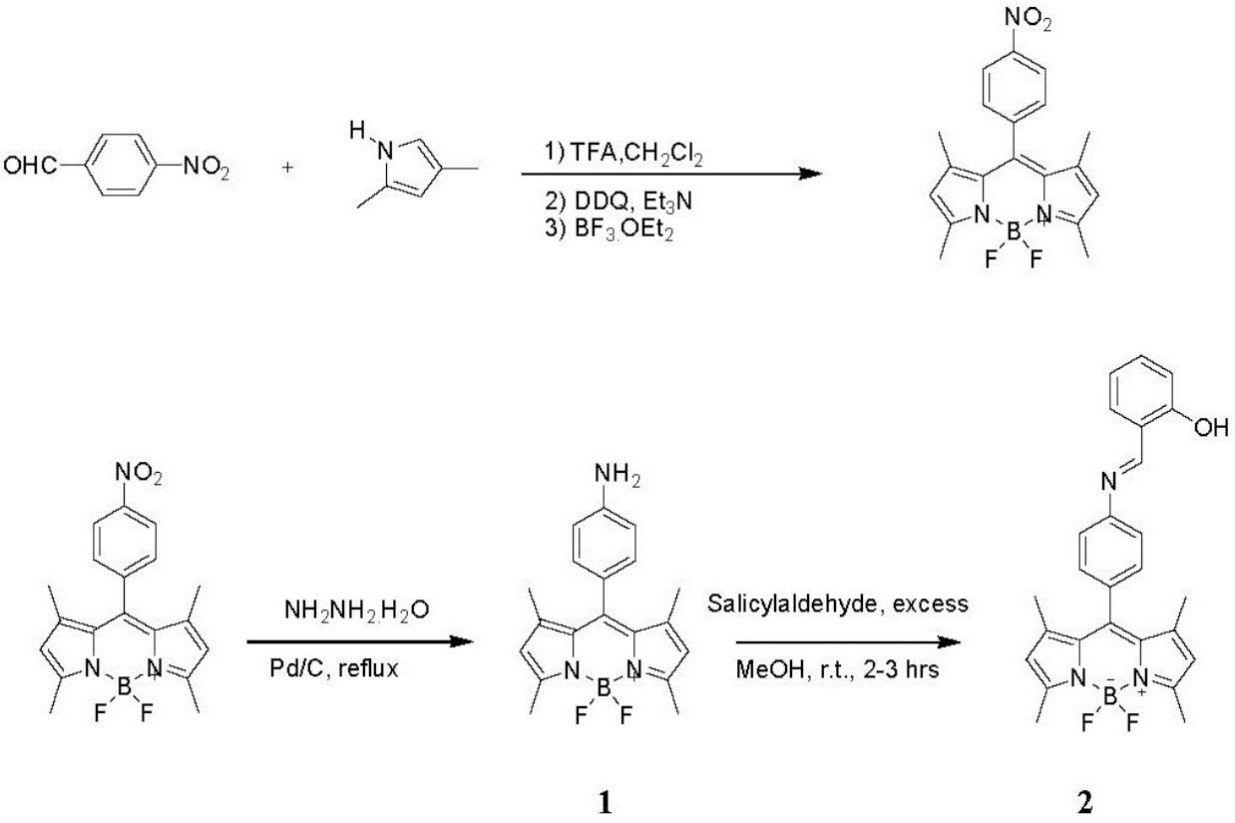
Synthesis of compounds **1** and **2**.

**Scheme 2.**
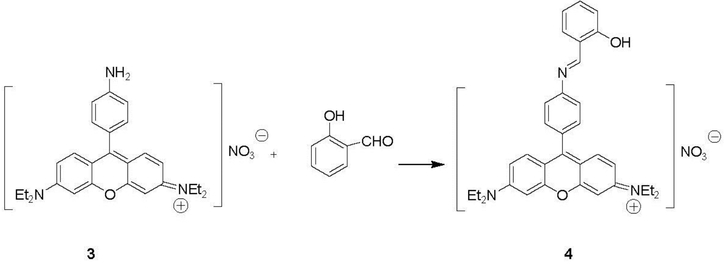
Synthesis of compounds **3** and **4**.

The synthetic routes that were used for the preparation of sensors **1** and **3** and the model compounds **2** and **4** are shown in Schemes 1 and 2. Nitro-BDP was synthesized from 2,4-dimethylpyrrole and p-nitrobenzaldehyde in one-pot reaction according to Imohori *et al*. [10] Reduction of nitro-compound was performed by refluxing hydrazine hydrate with Pd/C for 30 min, which cleanly gave a very good yield of amino-BDP product **1**. Combining salicylaldehyde with compound **1** in methanol at room temperature produced the BDP-imine product **2** (Scheme 1).

The rosamine sensor was synthesized according to Clunas *et al.* [11] (Scheme 2), with modifications noted in the experimental section. The intermediate product of nitrorosamine compound was synthesized by heating two equivalents of m-diethylaminophenol with one equivalent of 4-nitrobenzaldehyde. After purification with flash column chromatography, the rosamine chromophore was created in a single pot. Sulfuric acid was added to water and mixed with compound. The reaction mixture was then heated to 70 °C under nitrogen for 20 h and then at 90 °C for 29 h. Finally, nitric acid salt of the final crude nitro-substituted product was obtained in a very good yield and was isolated via filtration rather than column chromatography. Reduction of nitrorosamine was achieved by refluxing the intermediate with hydrazine hydrate and Pd/C in ethanol for 30 min. Compound **3** was isolated without any further purification, so the procedure is very easy to handle and amenable to scale up. Compound **4** was prepared in the same manner as compound **2**.

**Figure 2.**
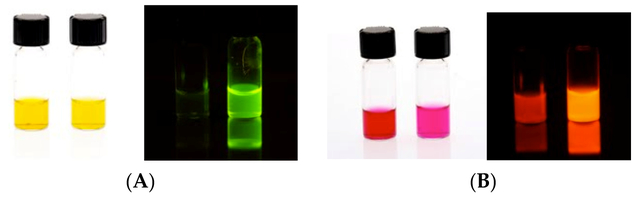
(**A**) Fluorescence of BDP compounds **1** and **2** under day (**left**) and UV fluorescent light (**right**) in methanol. (B) Fluorescence of rosamine compounds **3** and **4** under day (**left**) and UV fluorescent light (**right**) in methanol.

Figure 2 demonstrates visually the large increase in fluorescence that results when BDP **1** and rosamine **3** react with an aldehyde to form an imine. Fluorescence from BDP amine **1** is not visually detectable, while bright fluorescence is clearly seen in the solution of **2**. A smaller visual difference is seen with rosamine fluorophore **3** and its imine **4**, but the difference is clear.

Quantification of the fluorescence properties of the probes and their imines was performed and is shown in Tables 1 and 2. As expected, imine formation has little effect on the absorption and emission energies of the chromophores. Due to the presence of methyl groups at the C-1 and C-7 positions of BDP, the pi-systems of the amino benzene moiety and BDP moiety are twisted and conjugatedly uncoupled [8,9]. Therefore, the aromatic ring does not affect the energies of the electronic transitions in the BDP chromophore. The 75-fold increase in quantum yield in methanol without a change in absorption or emission maxima supports this mechanism for the fluorescence increase (Table 1). Interestingly, the difference in fluorescence in dioxane is less than that in methanol. We hypothesize that the hydrogen bonding ability of methanol serves to dampen the fluorescence of the parent amine but much less so with the imine.

**Figure 3.**
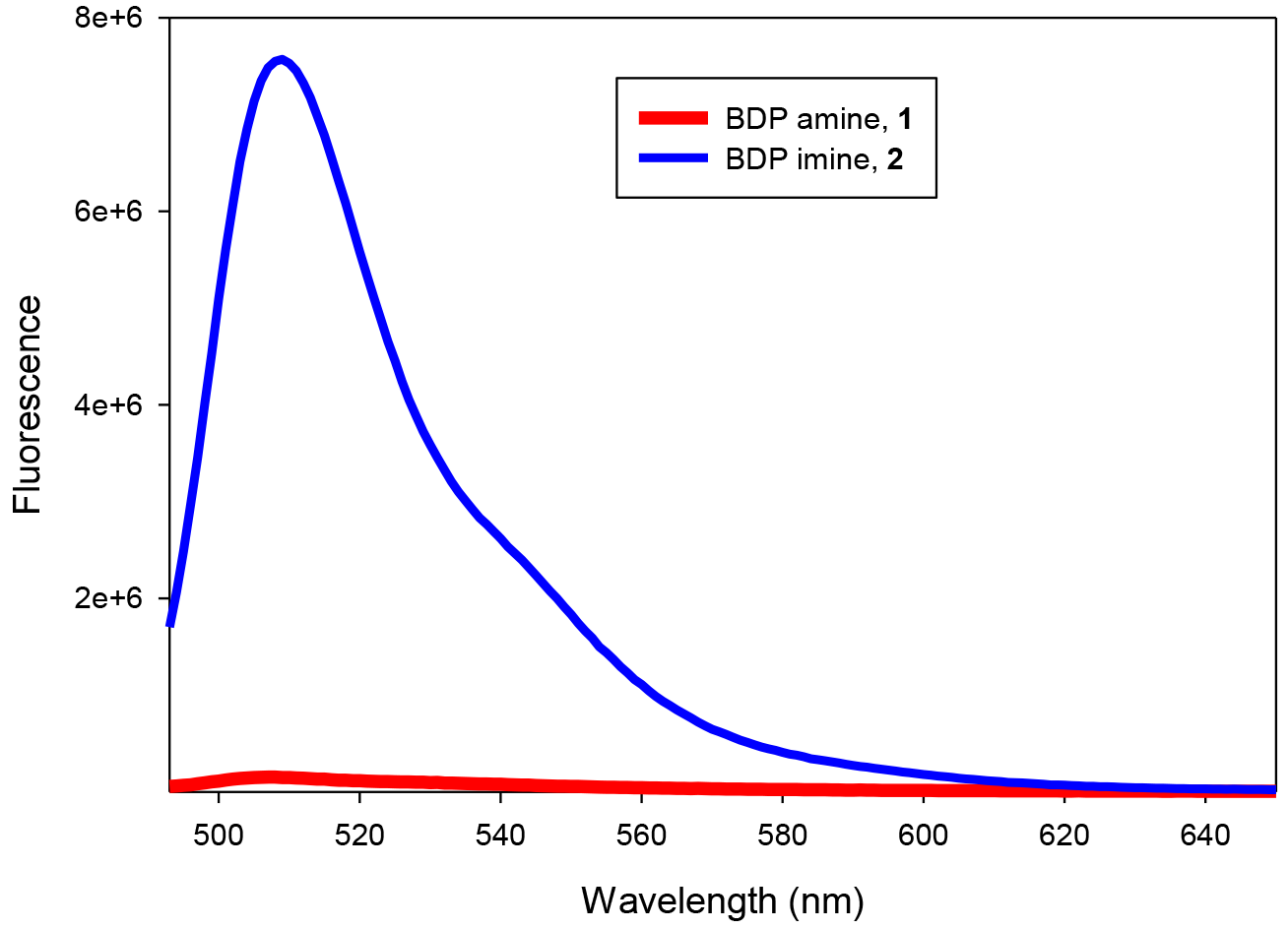
Emission spectra of compound BDP amine **1** (**red**) and its BDP imine **2** (**blue**) in methanol.

The rosamine probe shows the same behavior: increase in quantum yield without a change in absorption and emission energies (Table 2). The behavior is in accordance with the mechanism proposed by Urano *et al.* [11] This group studied the effect of the 2’ substituent on fluorescein fluorescence and showed that the fluorescein molecule could be understood as a directly linked donor-acceptor system, in which the aromatic ring is the donor and the xanthene ring is the acceptor. The electron donating group, amine, on the para position on phenyl group lowers the LUMO energy of the benzene moiety enough to quench the fluorescence of the xanthene. However, imine formation increases the energy of the aromatic LUMO sufficiently to prevent the electron transfer, and, therefore, the xanthene fluoresces. We note in our system that the difference in quantum yield is smaller for the rosamine fluorophore than the BDP fluorophore, but the difference is still substantial (10-fold) (Figure 3). A possible solvent effect could not be assessed because both **3** and **4** are insoluble in dioxane

## 3. Experimental Procedures

### 3.1. General Procedures

^1^H, ^13^C, ^11^ B NMR spectra were recorded on instruments operating at a frequency of 360 MHz. ^1^H-NMR spectra were referenced to CDCl_3_ (7.26 ppm). ^13^C-NMR spectra were referenced to the CDCl_3_ (77.00 ppm). ^11^B NMR spectra were referenced to BF_3_.OEt_2_ (0 ppm). Chemical shift multiplicities are reported as s = singlet, d = doublet, t = triplet, q = quartet and m = multiplet. Flash column chromatography was performed using Baker silica gel 60–200 mesh or 200–400 mesh. All flash column chromatography purifications were done by using TLC solvent conditions which were indicated in the experimental part. Absorption spectra of all compounds were obtained by using a Hewlett-Packard 8453 diode array absorption spectrophotometer. Fluorescence emission spectra for these molecules were measured using Spex FluoroMax-3 spectrofluorometer. Reactions were monitored by thin layer chromatography using TLC plastic sheets, silica gel 60 F_254._

### 3.2. Materials

All the solvents were degassed with argon before use. Dry methanol and dioxane were purchased from Acros and Aldrich. Other solvents and reagents were also obtained from Acros or Aldrich and were used as received. Deuterated solvents were obtained from Cambridge Isotope Laboratories. All other chemicals are commercially available and used without further purification.

### 3.3. Absorption and Fluorescence Analysis

UV-Vis spectroscopy is used for observing the reaction of BDP and rosamine-amine with aromatic aldehyde. Salicylaldehyde was frequently used as aromatic aldehyde.

Fluorescence studies were performed on a Spex FluoroMax-3 spectrofluorometer. The relative fluorescence quantum yields (ϕ_F_) were determined in dilute solutions with an absorbance below 0.1 at the excitation wavelength. Quinine sulfate in 0.1 M H_2_SO_4_ (λex = 347 nm) was used as a standard, which has a quantum yield of 0.57. Solvents were dried before use. The slit width was 2 nm for both excitation and emission. The relative quantum yields were obtained by calculating the area under corrected emission spectrum of the sample and comparing these areas with the area under corrected emission spectrum of standard solution of quinine sulfate. Correction for the refractive index was also applied. All spectra were recorded at 23 °C. All the areas under the fluorescence bands were calculated by using SigmaPlot 10.0 program. The relative quantum efficiencies of fluorescence were obtained with the following equation:

$$\phi_{\text{F}}{}^{\text{ sample }}=\phi_{\text{F standard }}\times ({\text{F}}_{\text{sample}}{\text{/F}}_{\text{standard}})\times ({\text{A}}_{\text{standard}}/{\text{A}}_{\text{sample}})\times ({\text{A}}_{\text{standard}}/{\text{A}}_{\text{sample}})(\eta^{2}{}_{\text{sample}}/\eta^{2}{}_{\text{standard}})$$

where F denotes the area under the fluorescence band, A denotes the absorbance at the excitation wavelength, and η denotes the refractive index of the solvent.

### 3.4. Synthesis of Aromatic Boron Dipyrromethenes (BDP) Derivatives

#### 1,3,5,7-Tetramethyl-8-(4’-salicylideneaminephenyl)-4,4-difluoro-4-bora-3a,4a-diaza-s-indacene (2)

Compound **1** (20 mg, 0.06 mmol) in methanol was purged with N_2_ for 10 min. Salicylaldehyde (63 μL, 1.2 mmol) was added and stirred at room temperature for 2 h. The solution was evaporated with N_2_ and applied to a silica gel flash column chromatography. Elution with 1:1 EtOAc :hexane *v/v* yielded **2** (11 mg, 42 %); ^1^H-NMR (360 MHz, CDCl_3_): *δ* 1.44 (s, 6H, -CH_3_), 2.55 (s, 6H, -CH_3_), 5.99 (s, 2H, -CH pyrrole), 6.91–7.08 (m, 3H, aromatics), 7.31–7.48 (m, 5H, aromatics), 8.71 (s, 1H, -CH). ^13^C-NMR (360 MHz, CDCl_3_): *δ* 14.5 (2), 14.9, 117.3, 118.9, 121.2, 121.9, 129.2, 130.8, 131.2, 131.4, 132.4, 133.5, 142.9, 149.1, 155.6, 161.2, 163.5. ^11^B NMR (360 MHz, CDCl_3_) δ 0.55 (t, *J* = 30.5 Hz). MS (ESI) M^+^ 443.3 found 444.

#### (5,5’-Bis-diethylamino-2,2’-(4-nitrobenzilidine)-di-phenol) intermediate^11^

3-diethylaminophenol (3.00 g, 18.18 mmol) was added to methanol (30 mL). Hydrochloric acid (1.04 mL) was then added to the mixture. 4-nitro-benzaldehyde (1.37 g, 9.09 mmol) was added to the reaction mixture. The reaction mixture heated to 40 °C for 18 h and the 50 °C for 24 h after which TLC analysis (1:1 EtOAc/Hexane (R_f_ = 0.3)) showed the reaction to be almost complete. The reaction mixture was poured into water (40 mL) and the Ph of the resulting mixture basified by the addition of an aqueous solution of saturated sodium bicarbonate. The mixture was extracted with dichloromethane (3 × 30 mL) and the combined extracts dried with sodium sulfate. The solvent was removed under reduced pressure to yield dark red oil. Column chromatography (1:1EtOAc/Hexane) gave the product as an orange-red solid (2.84 g, 69%)

#### 3,6-Bis-diethylamino-9-(4-nitrophenyl)xanthylium nitrate^11^ –

Sulfuric acid (1.2 mL) was added to water (120 μL) and cooled to 5 °C in ice. Previous intermediate (400 mg, 0.863 mmol) was added and the mixture heated to 70 °C under N_2_ for 20 h and then at 90 °C for 29 h. The resulting solution was cooled to 6 °C in ice and water (4 mL) added. The mixture was neutralized by the addition of sodium hydroxide (20%) whilst maintaining a reaction temperature of less than 16 °C. Hydrochloric acid (1.2 mL) was added and the reaction mixture stirred at 19 °C for 30 min under N_2_. FeCl_3_.6H_2_O (467 mg, 1.73 mmol) in water (4 mL) was added and the mixture heated to 88 °C for 3 h in air. The reaction was allowed to cool to 20 °C overnight. Water (4 mL) and HNO_3_ (dropwise, 70%) was added slowly until a solid precipitated. The solution was kept in 4 °C for overnight. The resulting green/purple precipitate was collected by suction filtration and dried under vacuum.

#### 3,6-Bis-diethylamino-9-(4-aminophenyl)xanthylium nitrate^11^

A suspension of previously synthesized nitro-compound (100 mg, 0.22 mmol) in EtOH (40 mL) was purged with N_2_ for 10 min. Hydrazine monohydrate (0.15 mL) and 10% Pd/C (23 mg, 0.1 equiv) were added, and the mixture was refluxed under nitrogen for 30 min. Then, Pd/C was removed under vacuum filtration, and the solvent evaporated under reduced pressure to afford a pink solid (88 mg, 98%): ^1^H-NMR (360 MHz, CD_3_OD) *δ* 7.65 (d, 2H, *J =* 8.97 Hz), 7.26 (d, 2H, *J =* 8.97 Hz), 7.05–7.12 (dd, 2H, *J =* 2.13 Hz), 6.93 (m, 4H, aromatics), 3.68 (q, 8H, CH_2_), 1.32 (t, 12H, CH_3_).

#### 3,6-Bis-diethylamino-9-(4-salicyliminephenyl)xanthylium nitrate (3)

A suspension of previously synthesized amino-compound (100 mg, 0.22 mmol) in EtOH (40 mL) was purged with N_2_ for 10 min. Salicylaldehyde was added, and the mixture was stirred under N_2_ for 30 min. Then, the solvent evaporated under reduced pressure to afford a pink solid. Imine product was not purified due to reversibility of imine in methanol and imine formation was only monitored in NMR tube by NMR spectroscopy. ^1^H-NMR (360 MHz, CD_3_OD) *δ* 8.80 (s, CH), 8.50 (s, 1H, OH), 7.60–7.27 (m, aromatics, 8H), 7.08–6.87 (m, 6H, aromatics), 3.66 (q, 4H, CH_2_), 1.31 (t, 6H, CH_3_).

### 3.5. Absorption and Fluorescence Analysis

UV-Visible spectra were obtained using an HP model 8453 UV-Vis spectrophotometer. Fluorescence spectra were measured on Jobin Yvon Fluoromax 3 spectrophotometer at 23–25 °C. The solvents for spectroscopic studies were distilled and degassed with argon before each experiment.

## 4. Conclusions

BDP and rosamine amines were designed and characterized as turn off/on fluorescent probes for the detection of aldehydes in organic solvents. As expected with a PeT mechanism, the amine containing molecules displayed low fluorescence. Conversion of amine group to an imine group successfully blocked the d-PeT process and enabled us to attain efficient turn off/on switching with the appropriate reaction site of the probe. Therefore, these dyes should be very useful tools for selective fluorescent monitoring of aldehydes in organic solvents. These molecules and this principle should have multiple applications in new sensor technology.

## Figures and Tables

**Table 1.** Summary of spectral properties of boron dipyrromethene (BDP) amine/imine system.

Compound	Solvent	λex_max_ (nm)	λem_max_ (nm)	Ф_*F*_^b^_(at 23 °C)_
1	Dioxane	491	507	0.15
1	Methanol	488	503	0.002
2	Dioxane	492	509	0.5
2	Methanol	488	507	0.27

**Table 2.** Summary of spectral properties of rosamine amine/imine system.

Compound	Solvent	λex_max_ (nm)	λem_max_ (nm)	Ф_*F*_^b^_(at 23 °C)_
3	Methanol	548	576	0.006
4	Methanol	548	578	0.06
